# Real-world evidence for regulatory decision-making: updated guidance from around the world

**DOI:** 10.3389/fmed.2023.1236462

**Published:** 2023-10-30

**Authors:** Leah Burns, Nadege Le Roux, Robert Kalesnik-Orszulak, Jennifer Christian, Jennifer Dudinak, Frank Rockhold, Sean Khozin, John O’Donnell

**Affiliations:** ^1^Worldwide Health Economics and Outcomes Research, Bristol Myers Squibb, Princeton, NJ, United States; ^2^Regulatory Intelligence, Bristol Myers Squibb, Boudry, Switzerland; ^3^Global Regulatory Sciences, Bristol Myers Squibb, Princeton, NJ, United States; ^4^Target RWE, Durham, NC, United States; ^5^Global Regulatory Sciences, Bristol Myers Squibb, Summit, NJ, United States; ^6^Department of Biostatistics and Bioinformatics, Duke Clinical Research Institute, Durham, NC, United States; ^7^Massachusetts Institute of Technology, Cambridge, MA, United States

**Keywords:** real-world evidence, real-world data, regulatory decision-making, real-world evidence regulatory frameworks, real-world data quality methods, real-world study methods, health technology assessment

## Abstract

Leveraging the value of real-world evidence (RWE) to make informed regulatory decisions in the field of health care continues to gain momentum. Improving clinical evidence generation by evaluating the outcomes and patient experiences at the point-of-care would help achieve the ultimate aim of ensuring that effective and safe treatments are rapidly approved for patient use. In our previous publication, we assessed the global regulatory landscape with respect to RWE and provided a review of the regional availability of frameworks and guidance through May 2021 on the basis of 3 key regulatory elements: regulatory RWE frameworks, data quality guidance, and study methods guidance. In the current review, we have updated and elaborated upon recent developments in the regulatory RWE environment from a regional perspective under the same 3 regulatory elements stated above. In addition, we have also included a new category on procedural guidance. The review also discusses the perceived gaps and potential opportunities for future development and harmonization in this field to support framework establishment in regions without pre-existing RWE policies. Additionally, the article reviews current developments of health technology assessment (HTA) bodies pertaining to RWE and discusses the status of evidentiary alignment among regulators and HTA agencies.

## Introduction

Real-world evidence (RWE) continues to receive global attention with growing use in supporting regulatory decision-making for drugs and biologics. Accordingly, regulatory bodies across the globe have continued to release several guidance documents on RWE. Still, the use of RWE in regulatory decision-making for effectiveness remains an emerging area, where in addition to guidance, there continues to be a role for the experience from precedents and pilots in informing best practices.

In our previous publication, we described and compared regulatory RWE frameworks and guidance documents for drugs and biologics from different countries around the world, available through May 2021, highlighting key areas for convergence, as well as gaps in the evidence generation process (1). We recommended further harmonization of guidance across geographies, including the encouragement of continued cross-country and cross-stakeholder collaboration, and standardizing the regulatory submission process for RWE, including the development of real-world data (RWD) standards (1). Additionally, we also recommended the development of further guidance on RWE trial design (e.g., randomized controlled registry studies, pragmatic trials, and hybrid studies) (1).

Since then, many regulatory authorities have moved beyond general frameworks to release several RWE guidance documents. This review discusses new regulatory guidance documents published from June 1, 2021 to January 31, 2023. These have been systematically organized and described under 3 main categories: regulatory RWE frameworks, RWD quality guidance, and real-world study methods guidance (1). Further, we have also included a fourth ancillary category on procedural guidance, considering the content of some recently released guidance pertaining to submission of RWE documents and related processes. Additionally, this review discusses the harmonization efforts and challenges facing regulators and health technology assessment (HTA) bodies on the alignment of the use of RWE for decision-making.

## Methods

We performed a targeted literature search of all relevant articles from June 1, 2021 to January 31, 2023 related to regulatory RWE frameworks and policies, RWD data quality methods, and real-world study methods. An initial search was performed on all International Coalition of Medicines Regulatory Authorities (ICMRA) member sites for relevant guidance and reports based on the search parameters (2). Additional literature searches were conducted on databases (PubMed, Google) using specific keywords (real-world evidence; real-world data; regulatory guidance; regulatory guidance and RWE) to check for relevant academic publications, white papers, and/or news reports. Subsequently, cited references from the previous publication were reviewed and checked for updates within the current period of study to ensure that all relevant publications were captured. The selected articles were organized for qualitative synthesis under the following subheadings: the 3 key regulatory elements namely, regulatory RWE frameworks, RWD quality guidance, and real-world study methods guidance; and a fourth category on procedural guidance. An additional search on relevant HTA guidance related to RWE released in the current search period was also performed. The detailed methodology has been presented in Table 1.

**Table 1:** Methodology.

RWE and regulatory decision-making (Updates from June^**^ 2021 to January 2023)	
Literature search	Screening parameters: Relevant articles from June 1, 2021 to January 31, 2023 Topic of article: Regulatory frameworks and policies, data quality methods, study methods, and procedural guidance
Step 1	Initial search performed on all ICMRA member sites for relevant guidance and reports based on the screening parameters (2)
Step 2	Additional literature searches were conducted (PubMed, Google) using specific keywords (real-world evidence; real-world data; regulatory guidance; regulatory guidance and RWE) to check for relevant academic publications, white papers, and news reports
Step 3	Reviewed cited references from previous publication and checked them for updates within current time period to ensure that all relevant publications were captured
Selected articles for qualitative synthesis under: Regulatory frameworks and policies (*n* = 32)^*^ Data quality methods (*n* = 20)^*^ Study design methods (*n* = 10)^*^ Procedural guidance (*n* = 5)	

*Indicates that the articles are not exclusive and can fall under more than 1 subheading. **Please note that the publication date of 1 article (3) included in the current manuscript precedes the search period. Additionally, 5 HTA documents (guidance/frameworks/initiatives) related to RWE were also identified in the literature search.

## Global RWE regulatory environment

**Figure 1:**
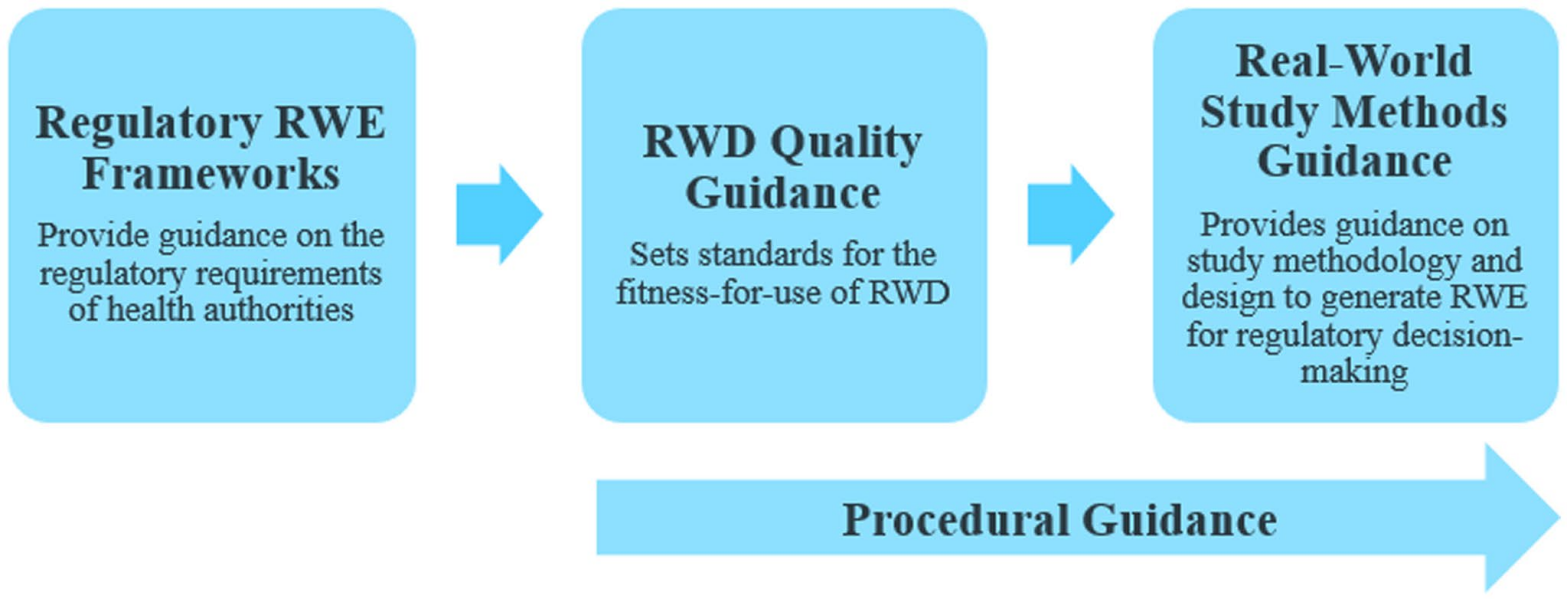
Key regulatory real-world evidence (RWE) elements. RWD, Real-world data.

**Figure 2:**
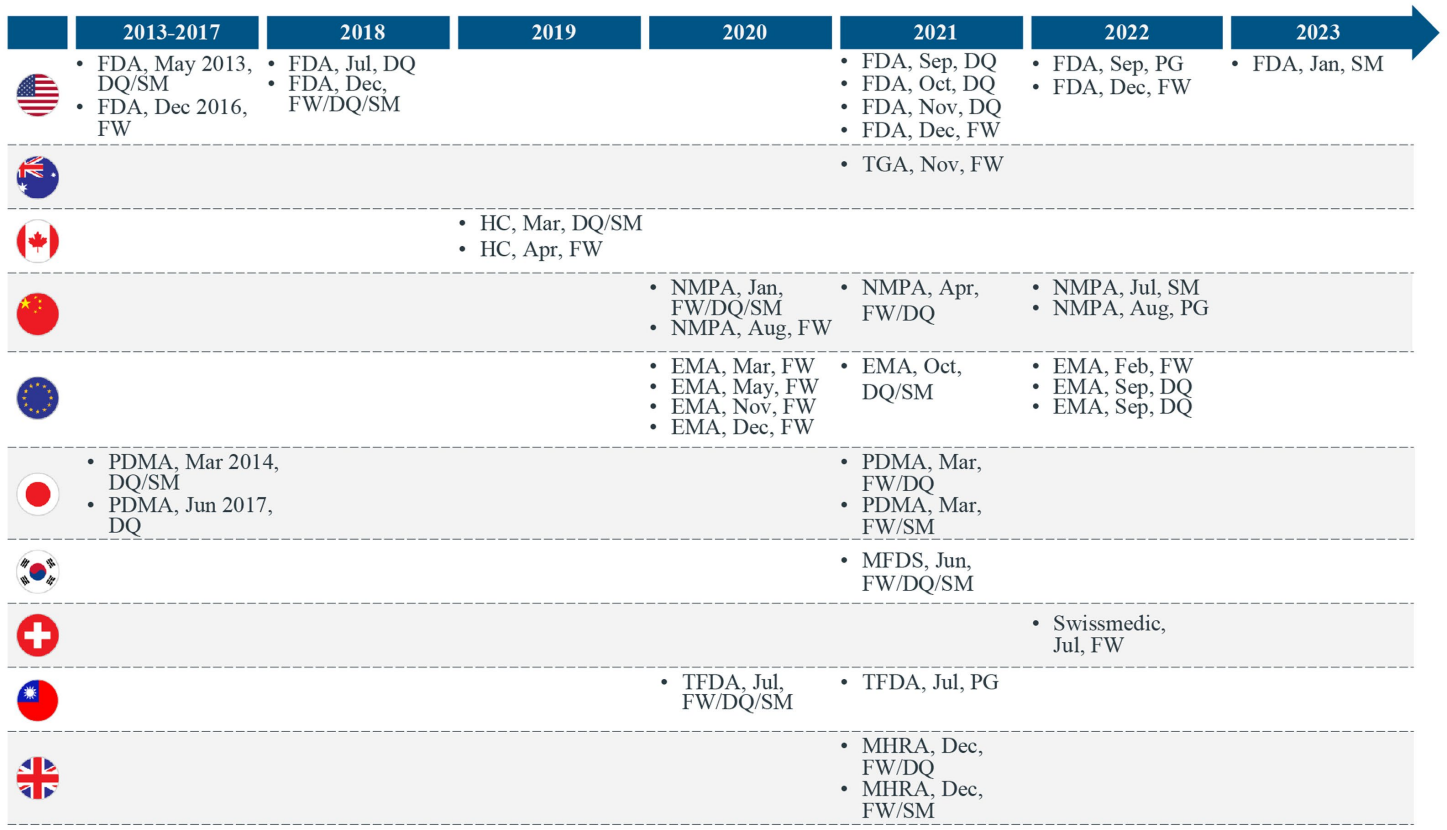
Trends in regulatory real-world evidence (RWE) guidance documents released across the world. EU, European Union; FDA, Food and Drug Administration; HC, Health Canada; FW, Framework; DQ, Real-world data quality guidance; MHRA, Medicines and Healthcare products Regulatory Agency; MFDS, Ministry of Food and Drug Safety; NMPA, National Medical Products Administration; PG, Procedural Guidance; PMDA, Pharmaceuticals and Medical Devices Agency; SM, Real-world study methods guidance; TFDA, Taiwan Food and Drug Administration; TGA, Therapeutic Goods Administration.

In our previous publication (1), we proposed that countries leveraging RWE for making regulatory decisions for drugs and biologics were trending toward a stepwise approach for developing the following 3 key regulatory elements: (a) regulatory RWE frameworks, (b) RWD quality guidance, and (c) real-world study methods guidance (Figure 1). With the recent RWE guidance documents released through January 2023, we have continued to observe this trend. Many major regulatory authorities have now shifted from the previously released initial frameworks or position papers to issuing fully detailed practical guidance documents on RWE in regulatory decision-making. Most of these guidance documents discuss data quality and standards (Figure 2). Regulatory authorities have also begun to release guidance on study methodology for different real-world study designs (Figure 2). In addition, there are a few guidance documents focused on the procedural aspects of engaging with regulatory agencies to discuss RWE and submit documents containing RWD/E (Figures 1, 2). Overall, the extent to which the above stepwise criteria have progressed (i.e., some countries may adopt existing frameworks instead of developing their own) indicates how advanced the RWE environment is as well as the feasibility of using such evidence for regulatory decisions in a specific location.

### Updates on regulatory RWE frameworks across the globe

Regulatory RWE frameworks in a given region can help provide an important grounding on the accepted RWE use during the life cycle of a product, including any pre-marketing activities and post-marketing labeling changes for safety and effectiveness. They may also provide clarification on the plans of regulatory agencies regarding the future release and adoption of more detailed guidance. In the last review, up to May 2021, most of the major regulatory agencies including the United States Food and Drug Administration (US FDA), European Medicines Agency (EMA), Health Canada, Medicines and Healthcare products Regulatory agency [MHRA, United Kingdom (UK)], Pharmaceuticals and Medical Devices Agency (PMDA, Japan), National Medical Products Administration (NMPA, China), and Taiwan Food and Drug Administration (TFDA) had released frameworks on RWE (1).

Since then, many additional regulatory agencies have also released initial frameworks/positions, including Swissmedic (Switzerland), Ministry of Food and Drug Safety (MFDS, South Korea), and Therapeutic Goods Administration (TGA, Australia). Table 2 summarizes the regulatory RWE frameworks available across the globe, grouped by North America, Europe, and Asia-Pacific regions.

**Table 2:** Regulatory RWE frameworks and policies in North America, Europe, and Asia-Pacific.

Regulatory body	Framework or policy	
	Legislation	RWE frameworks
**North America**		
US Food and Drug Administration (FDA)	21st Century Cures Act (2016) (4) **PDUFA VII (2022)** (5)	FDA RWE Framework (2018) (6) **Draft guidance: Considerations for the use of real-world data and real-world evidence to support regulatory decision-making for drug and biological products (2021)** (7) (New)
Health Canada (HC)	NA	Optimizing the use of RWE to inform regulatory decision-making (2019) (8)
**Europe**		
European Medicines Agency (EMA) /European Commission (EC)	Pharmaceutical Strategy for Europe (Ongoing) (9) **Data Act (2022)** (10)	Regulatory science to 2025 (2020) (11) European medicines agencies network strategy to 2025 (2020) (12) HMA/EMA Big data task force recommendations and big data steering group (2020) (13, 14)
Medicines and Healthcare products Regulatory Agency (MHRA), United Kingdom (UK)	NA	**MHRA guidance on the use of real-world data in clinical studies to support regulatory decisions (2021)** (15) (New) **MHRA guideline on randomized controlled trials using real-world data to support regulatory decisions (2021)** (16) (New)
Swissmedic, Switzerland	NA	**Swissmedic position paper on the use of real-world evidence (2022)** (17) (New)
**Asia-Pacific**		
National Medical Products Administration (NMPA, China)	NA	Guidelines for Real-World Evidence to Support Drug Development and Review (Interim) (2020) (18) Technical Guidelines for Real-World Research Supporting the R&D and Review of Pediatric Drugs (Trial) (2020) (19) Guiding principles of real-world data used to generate real-world evidence (Trial) (2021) (20)
Taiwan Food and Drug Administration (TFDA)	NA	Basic considerations for real-world evidence supporting drug research and development (2020) (21)
Pharmaceuticals and Medical Devices Agency (PMDA, Japan)	NA	Basic principles on utilization of registry for applications (2021) (22) Points to consider for ensuring the reliability in utilization of registry data for applications (2021) (23)
Ministry of Food and Drug Safety (MFDS, South Korea)	NA	**Medical information database studies guideline (2021)** (24) (New)
Therapeutic Goods Administration (TGA, Australia)	NA	**Real-world evidence and patient-reported outcomes in the regulatory context (2021)** (25) (New)

RWE, Real-world evidence; NA, Not applicable. Bold font denotes new guidance updates from June 2021 to January 2023.

### North America

In the US, passing of the Prescription Drug User Fee Act (PDUFA) VII (5) has added further clarity regarding RWE use in regulatory decision-making into the legislation, including the launch of FDA’s Advancing Real-World Evidence (RWE) Program (26, 27), a pilot program seeking improvement in the quality and acceptability of RWE-based approaches to support new intended labeling claims or satisfy post-approval study requirements. In addition, the FDA clarified the applicability of Investigational New Drug regulations to non-interventional real-world studies in its draft guidance on considerations for the use of RWD and RWE to support regulatory decision-making released in December 2021 (7).

### Europe

The European Union (EU) has seen further advancement of legislation relevant to RWE use in drugs and biologics. Notably, the European Commission (EC) presented a legislative proposal in May 2022 for the establishment of a European Health Data Space (EHDS) to harness the maximum potential of digitalized health data (28). The EHDS would be a health-based ecosystem where rules, standards, practices, and infrastructures can be brought under a common governance framework via the establishment of a secure centralized data system. It aims to streamline the processes of primary use of data, i.e., increased digital access of electronic health record systems to patients, and to facilitate secondary data use among researchers, policymakers, and regulators. In July 2022, the EC chose a consortium led by the French Health Data Hub to launch a pilot project for the EHDS (29). The pilot aims to address the obstacles in accessing health data across Europe, such as data fragmentation, and to set up a concrete foundation for the EHDS over the next 2 years. The practicality of data reuse from different European countries is being tested via use cases spanning a multitude of research themes including infectious disease surveillance and disease care pathways (30).

The EHDS further builds on the Data Act that was adopted by the EC on February 23, 2022 (10). It is a regulation for harmonized rules on fair data access and use and constitutes a key pillar of the European data strategy. The Heads of Medicines Agencies (HMA)/EMA Big Data Steering Group (BDSG) also announced the launch of the Data Analysis and Real World Interrogation Network (DARWIN) EU platform (13, 31, 32), which is expected to act as a pathfinder to coordinate with the EHDS in order to enable EMA and national competent authorities to use RWD from the DARWIN EU network for regulation of medicinal products throughout their lifecycle. DARWIN EU constitutes a single, centralized data, expertise, and services network for improved decision-making via timely and reliable evidence generation from real-world healthcare databases across the EU (33). In January 2023, the EMA selected the first set of data partners, from different countries across the EU, to collaborate with the DARWIN EU platform (34). In addition, the EU Pharmaceutical Strategy (9),^1^ which included a plan to update the legislation for incorporating new approaches of evidence generation and assessment, such as the analysis of big data and RWD for supporting new medicine authorization, is continuing efforts to streamline processes. Several workshops and stakeholder engagement initiatives also continue to be organized to drive forward the other recommendations of the HMA/EMA joint Big Data Task Force (35–38). Acting on the focus areas set forth by the Network Strategy to 2025 and BDSG workplan 2021–2023, the BDSG has continued to make significant progress in the transformation to a data-driven regulatory ecosystem throughout 2021 and 2022 (12, 39–41). The HMA-EMA BDSG published its updated workplan (3rd in the series) in July 2022 that outlines key deliverable actions in relation to the 10 recommendations, including DARWIN EU, data quality, data discoverability, and EU network skills for 2022–2025, so as to further the effective integration of data analysis into medicinal product evaluation by regulators (42). These will be discussed in the subsequent sections.

Further, at the committee level, the Committee for Medicinal Products for Human Use (CHMP), the Committee for Advanced Therapies (CAT), the Pharmacovigilance Risk Assessment Committee (PRAC), the Pediatric Committee (PDCO), the Committee for Orphan Medicinal Products (COMP), and the Scientific Advice Working Party with the Biostatistics Working Party and others, play a key role in contributing to the EU regulatory guidelines and running their own pilots on the use of RWE to inform the guidelines and where RWE may be most appropriate in their decision making. This is particularly important considering their role in scientific advice, designation, and review assessment including marketing authorization.

In the United Kingdom, the MHRA released the final guidance on the use of RWD in clinical studies to support regulatory decisions, which introduces the MHRA’s overall RWD guideline series and covers general principles for the use of RWE in the United Kingdom (15, 16).

In Switzerland, Swissmedic released a position paper on the use of RWE, which clarified that although the agency accepts the use of RWE as supportive evidence to complement clinical trial data, new marketing applications based on RWE only are currently not accepted (17). As per legislation, the market authorization documentation for applications to Swissmedic must include the results of Good Clinical Practices (GCP)-compliant clinical trials (17).

### Asia-Pacific

In South Korea, the MFDS released an RWE guideline on medical information database studies (24). Additionally, the MFDS and the Korean Academy of Medical Sciences jointly held the 3rd Regulatory Science Innovation Forum, “Utilizing Big Data for Regulatory Science Decision-Making,” where the MFDS committed to innovation in drug data in 2022 to meet industry needs, including the re-assessment of the use of real data (43, 44).

The TGA in Australia published a report reviewing RWE use and patient-reported outcomes, which was based on a consultation conducted by the agency (25). This has prompted the TGA to plan implementation of a number of activities, including establishment of a central point for information regarding RWE on the TGA website and communicating when RWE is used for making regulatory decisions (25). The TGA has also adopted FDA’s definition of RWE (45).

### South America

In South America, although no formal RWE frameworks have been released, Anvisa, the Brazilian Health Regulatory Agency, allows the use of RWD/E in regulatory submissions. According to a survey of industrial stakeholders, the use of RWD/E by the pharmaceutical industry in Brazil has evolved, with a majority (76% of respondents) using RWE for supporting drug access and 56% (of respondents) using RWE for regulatory submissions to Anvisa (46). The growing use of RWE in regulatory decision-making across the globe was also discussed in a multi-part workshop in 2021 as part of the knowledge expansion (ECHO) program conducted by Anvisa and Interfarma (47). Further, the new regulatory framework approved by Anvisa in 2022 favors the registration of new and innovative drugs aligned with the international scenario, via input of technical data including RWD/E (48).

### Third-party initiatives

As a third-party initiative (Supplementary Table S1), the Duke-Margolis Center for Health Policy RWE Collaborative actively engages stakeholders to enhance RWE development and use with the following 2022 priorities: (a) regulatory acceptability of RWD/E, (b) learning health care systems: RWD for evidence-based decision-making and shared learning, (c) master RWE protocols, and (d) real-world efficacy: patient subgroups (49). The European Health Data and Evidence Network (EHDEN) Academy, launched as part of the Innovative Medicines Initiative’s (IMI’s) EHDEN project in April 2020, has been a resource that provides tools, skills, and methods training for researchers conducting real-world studies (50). In October 2022, EHDEN joined a European stakeholders’ consensus encouraging decision-makers to support the EHDS regulation and also proposed a set of recommendations for successfully achieving digital transformation across Europe (51). The GetReal Institute conducted its inaugural annual conference in March 2023 to keep the RWE community informed of global developments and the future direction of RWE (52, 53), providing a platform to highlight ongoing initiatives and emerging challenges as well as an opportunity for collaboration to address these challenges.

### Updates on RWD quality guidance

Once a country establishes a general RWE framework, developing guidance on the components of high-quality RWD and the key criteria for assessment of RWD quality is an important subsequent step to clarify regulatory standards and ensure that data are fit-for-use. In our last publication, the NMPA and PMDA had released dedicated guidance documents on data quality while regulatory agencies such as FDA, EMA, and TFDA had released guidance that included data quality among other topics (1).

Since then, additional regulatory agencies have released their own guidance documents dedicated to RWD quality, including the FDA and EMA. Multi-topic guidance which also touch upon data quality have also been released by EMA and the MHRA. Table 3 summarizes the regulatory RWD quality guidance available across the globe.

**Table 3:** Regulatory guidance on real-world data (RWD) quality in Asia-Pacific, North America, and Europe.

Regulatory body	Guidance documents
North America	
U.S. Food and Drug Administration	Best practices for conducting and reporting pharmacoepidemiologic safety studies using electronic healthcare data (2013) (54) Use of electronic health record data in clinical investigations (2018) (55) FDA RWE framework (2018) (6) **Draft guidance: Assessing electronic health records and medical claims data to support regulatory decision-making for drug and biological products (2021)** (56) (New) **Draft guidance: Data standards for drug and biological product submissions containing real-world data (2021)** (57) (New) **Draft guidance: Assessing registries to support regulatory decision-making for drug and biological products guidance for industry (2021)** (58) (New)
Health Canada (HC)	Elements of real-world data/evidence quality throughout the prescription drug product life cycle (2019) (59)
Europe	
European Medicines Agency (EMA, European Union)	**Guideline on registry-based studies (2021)** (60) (New) **Draft guidance: Good practice guide for the use of the metadata catalog of RWD Sources (2022)** (61) (New) **Draft guidance: Data quality framework for EU medicines regulation (2022)** (62) (New)
Medicines and Healthcare products Regulatory Agency (MHRA, United Kingdom)	**MHRA guidance on the use of real-world data in clinical studies to support regulatory decisions (2021)** (15) (New) **List of metadata for Real World Data catalogs (2022) (New)**
Asia-Pacific	
National Medical Products Administration (NMPA, China)	Guidelines for Real-World Evidence to Support Drug Development and Review (Interim) (Interim) (2020) (18) Guiding principles of real-world data used to generate real-world evidence (trial) (2021) (20)
Taiwan Food and Drug Administration	Basic considerations for real-world evidence supporting drug research and development (2020) (21)
Pharmaceuticals and Medical Devices Agency (PMDA, Japan)	Guidelines for conducting pharmacoepidemiological studies in drug safety evaluation using medical information databases (2014) (63) Basic policy on the use of medical information databases in post-marketing pharmacovigilance (2017) (64) Points to consider for ensuring the reliability in utilization of registry data for applications (2021) (23)
Ministry of Food and Drug Safety (MFDS, Korea)	**Medical information database studies guideline (2021)** (24) (New)

Bold font denotes new guidance updates from June 2021 to January 2023.

### North America

In the United States, the FDA released several draft guidance documents (7, 56–58) focusing specifically on data quality and data standards, including guidance on the use of electronic health records (EHRs)/claims data (56), use of registry data (58), and data standards for real-world datasets (57). Overall, the draft guidance documents, particularly the EHR/claims guidance, discuss detailed recommendations for data source selection, study variable definitions and validation, quality control/assurance processes, documentation, and data source information that should be included in the protocol and statistical analysis plan. These guidance documents reflect an effort by the agency to increase the rigor and amount of information received on the data source in industry RWE proposals. All these guidance documents are draft versions (or updated draft versions) that have received abundant public interest and feedback; the final versions following consideration of the feedback are expected later in 2023 (within 18 months of the closing period of prior comment, as stated in the 21st Century Cures Act).

In Canada, the draft guidance from CADTH published in November 2022 provides best practices for attaining the maximum level of transparency in reporting relevant and useful RWE during submissions to Canadian regulatory and HTA bodies (65). The draft was developed with support from Canadian and international RWE experts [Health Canada, FDA, and National Institute for Health and Care Excellence (NICE)], and includes a checklist of items that should be reported during submissions on aspects related to RWD quality and characterization (65).

### Europe

In the EU, the EMA, in collaboration with HMA and TEHDAS (Joint Action Toward the European Health Data Space), released a draft “Data Quality Framework,” which serves as a general guideline encompassing considerations on data quality across data types and regulatory submissions, including definitions for data dimensions and sub-dimensions, as well as their characterization and related metrics (39, 62). The EMA also released a draft “Good Practice Guide for the Use of the EU Metadata Catalog of RWD Sources” (61) to enable data discoverability. It provides recommendations on use of the agency’s real-world metadata catalog, which will replace the existing catalog of the European Network of Centers for Pharmacoepidemiology and Pharmacovigilance (ENCePP) by late 2023 (66–68). Further, the EMA released the final version of the “Guideline on Registry-Based Studies” (60), which discusses various data quality considerations for registry data sources, generally consistent with the draft version discussed in the previous publication (1). Further, in order to assess the utility of raw data (i.e., clinical study-derived individual patient data available in electronic structured format for analysis and visualization) in improving and accelerating the process of medicine evaluation, the EMA launched a pilot initiative in July 2022, which will run for 2 years and include about 10 regulatory procedures submitted to the EMA (68–70). As part of the pilot, the EMA has received an initial marketing authorization application, with raw data, for the treatment of a neurological disorder. The Danish Medicines Agency will be responsible for conducting the analysis and visualization of the data as part of a contract with EMA following a procurement procedure (37). Currently, applicants submit data in an aggregated format, which can hinder its analysis and slow down the process of evaluation (69, 70). This pilot initiative aligns with the priority recommendation of building network capability to analyze big data, as per the Big Data Task Force (69, 70). Overall, regulators in the EU are consistently striving toward enabling and supporting data harmonization to minimize the administrative efforts involved in receiving, processing, and reusing scientific data across the EU, as well as at a global level. Thus, based on stakeholder consultations, the European Medicines Regulatory Network (EMRN) Data Standardization Strategy was developed that aligns with the HMA-EMA joint Big Data Task Force recommendations (71). The strategy outlines key principles for defining, adopting, and implementing international data standards by the EMRN (71, 72).

In the United Kingdom, the MHRA released guidance on the use of RWD in clinical studies to support regulatory decisions (15). Beyond the general introductions to the guidance series, this guidance also includes a dedicated section on MHRA’s recommendations around RWD quality.

### Asia-Pacific

In South Korea, the RWE guideline on medical information database studies also discusses attributes related to RWD quality (24).

### Third-party initiatives

In addition to formal regulatory guidance, several third-party initiatives are also working to help provide standards and tools for improving RWD quality and access (Supplementary Table S1). The One Source Program, a collaborative initiative by researchers from the University of California, San Francisco-Stanford University Center of Excellence in Regulatory Science and Innovation, and the FDA, aims to develop tools and methods for automation of structured EHR data flow into external systems, so as to reduce operational costs, time, and improve data quality for clinical studies (73). The FDA Oncology Center of Excellence (OCE) started the Quality Characteristics and Assessment of Real-world Data (QCARD) initiative in 2022, in collaboration with the Reagan-Udall Foundation, to develop a standardized approach for RWD characterization and evaluation of oncology RWD quality for regulatory submissions (74). The Audit Readiness Tool by TransCelerate BioPharma Inc. intends to promote best practices for quality management of RWD, such as inspection readiness, which is suitable for regulatory decision-making (75).

The ICARE data® (Integrating Clinical Trials and Real-World Endpoints) project aims to support the collection of high-quality RWD, based on Minimal Common Oncology Data Elements (mCODE™), to enable clinical oncology research (76). The HARMONY (Healthcare Alliance for Resourceful Medicine Offensive against Neoplasms in Hematology) Big Data platform provides access to a data lake and advanced analytic tools for the accelerated development of more innovative and effective treatments against hematological malignancies (77). The Clinical Data Interchange Standards Consortium (CDISC) has successfully standardized clinical trial-based data for regulation on a global scale. CDISC standards are required for regulatory submissions to the FDA and PMDA (78). However, more effort toward the adoption of an RWD standard that is fit-for-purpose is warranted. There are several challenges with adopting such data standards for RWD, including: (a) complexity of data, (b) concerns due to data being originally collected for different purposes, (c) lack of awareness and incentives to adopt new standards, and (d) inadequate implementation support and training (78). Potential solutions might result from further collaboration with other standards development organizations, establishing controlled terminologies and models which can be used to represent data from varied sources, as well as the development of tools and training to support the RWD community (78). An example of such a tool can be found in the Registry Evaluation and Quality Standards (REQueST) Tool, from the EUnetHTA Joint Action 3, which promises to maximize the utilization of registry data in decision-making by regulatory as well as HTA bodies (79).

### Updates on study methods guidance

Regulatory authorities have now begun to address study design methods for RWE studies intended for regulatory decision-making. In the last review until May 2021, the MHRA had released a draft guidance on evaluating methods for a specific study design, randomized clinical trials (RCTs) incorporating RWE (80). Guidance documents discussing multiple topics including general study methods (among other topics) were also released by the FDA, EMA, NMPA, PMDA, and TFDA (1).

Since then, additional regulatory authorities, including the FDA and MFDS, have begun to release their own guidance documents, focusing on study methods for various real-world study designs. Updated or additional guidance on study methods have also been released by the EMA, MHRA, and NMPA. Table 4 summarizes the regulatory real-world study methods guidance available across the globe.

**Table 4:** Regulatory guidance on real-world study methods in Asia-Pacific, North America, and Europe.

Regulatory body	Guidance documents
North America	
US Food and Drug Administration (FDA)	Best practices for conducting and reporting pharmacoepidemiologic safety studies using electronic healthcare data (2013) (54) FDA RWE framework (2018) (6) **Draft guidance: Considerations for the Design and Conduct of Externally Controlled Trials for Drug and Biological Products (2023)** (81) (New)
Health Canada (HC)	Elements of real-world data/evidence quality throughout the prescription drug product life cycle (2019) (59)
Europe	
European Medicines Agency (EMA)	**Guideline on registry-based studies (2021)** (60) (New)
Medicines and Healthcare products Regulatory Agency (MHRA, United Kingdom)	**MHRA guideline on randomized controlled trials using real-world data to support regulatory decisions (2021)** (16) (New)
Asia-Pacific	
National Medical Products Administration (NMPA, China)	Guidelines for Real-World Evidence to Support Drug Development and Review (Interim) (2020) (18) **Draft guidance: Guiding Principles for Drug Real-World Research Design and Protocol Framework (2022)** (82) (New)
Taiwan Food and Drug Administration (TFDA)	Basic considerations for real-world evidence supporting drug research and development (2020) (21)
Pharmaceuticals and Medical Devices Agency (PMDA, Japan)	Guidelines for conducting pharmacoepidemiological studies in drug safety evaluations using medical information databases (2014) (63) Basic principles on utilization of registry for applications (2021) (22)
Ministry of Food and Drug Safety (Korea)	**Medical information database studies guideline (2021)** (24) (New)

Bold font denotes new guidance updates from June 2021 to January 2023.

### North America

In the US, the FDA released its draft guidance in 2023, “Considerations for the Design and Conduct of Externally Controlled Trials,” which includes recommendations for external controls consisting of other clinical trial data or RWD (81). Overall, the guidance discusses numerous study design and analysis considerations centered around commonly encountered challenges for this type of study design when intended to be used for regulatory purposes. The FDA is also expected to publish two additional draft guidance documents in 2023 on non-interventional studies and the use of clinical practice data in randomized clinical trials.

The CADTH draft guidance on reporting RWE also highlights methodological considerations for RWE studies and their submission to regulators and HTA bodies in Canada (65).

### Europe

In the EU, the EMA’s final “Guideline on Registry-Based Studies” includes methodology recommendations related to registry-based study designs, including expected protocol elements and areas of anticipated bias (60), and is generally consistent with the draft version of the guidance included in our prior review (1). The DARWIN EU also supports in making regulatory decisions by addressing specific questions through high-quality, non-interventional studies, including the development of scientific protocols, examination of relevant sources of data, and analysis and reporting of the results (31).

In the United Kingdom, the MHRA’s final “Guideline on Randomized Controlled Trials Using RWD to Support Regulatory Decisions” contains various methodology recommendations with regard to the appropriate design and operation of such studies (16), and is generally consistent with the draft version of the guidance described in our previous review (1).

### Asia-Pacific

In China, the NMPA’s Center for Drug Evaluation issued a draft guideline in 2022 dedicated to study methods and protocol development for any real-world study design, including observational studies, external controls, and pragmatic trials. The guidance emphasized the need for a strong scientific approach and also clarified the content/section expectations for real-world research protocols in China (82).

In South Korea, the MFDS also provides considerations for RWE study designs in its “Medical Information Database Studies Guideline,” including protocol and study report writing methods (24).

### Third-party initiatives

Apart from the formal regulatory agency guidance, several third-party initiatives have continued to work on topics concerning RWE study methods (Supplementary Table S1). The RCT DUPLICATE (Randomized, Controlled Trials Duplicated Using Prospective Longitudinal Insurance Claims: Applying Techniques of Epidemiology) initiative has been using RWD to reproduce RCTs to further inform when it is possible to use RWD successfully, the best methods to implement such studies, and the future of quality assurance processes. An interim report on the initial findings of this project has been published (3). The Duke-Margolis Center for Health Policy has also continued to release whitepapers and conduct workshops on point-of-care trials, i.e., RCTs using RWD, or pragmatic trials (83). Friends of Cancer Research has also been conducting a pilot program on developing real-world endpoints (84). The IMI’s GetReal Institute launched in April 2021 also provides RWE training through its GetReal Academy (53). With a mission to facilitate RWE adoption and implementation in healthcare decision-making across Europe, the institute works on three key focus areas: reducing barriers in secondary data use, bridging the gap between RWE and RCTs, and addressing evidence needs of decision-makers.

### Updates on procedural guidance

Several regulatory authorities have also released procedural guidance related to RWE (Table 5). The TFDA released a guideline for categorizing submissions as containing RWE for agency-tracking purposes (87), similar to the FDA’s draft guidance on the same topic released in 2019. The final version of the FDA guidance was released in late 2022 (85). Additionally, the NMPA released a draft guidance on meetings with CDE regarding RWE studies planned to support registrational applications (86). In Europe, the adoption of the Interoperable Europe Act by the EC in November 2022 was an important step to strengthen cooperation among EU member states and institutions for a secure cross-border exchange of data and its interoperability (88).

**Table 5:** Procedural guidance on real-world evidence (RWE) application submissions in Asia-Pacific and North America.

Regulatory body	Guidance documents
North America	
US Food and Drug Administration (FDA)	**Submitting documents using real-world data and real-world evidence to FDA for drug and biological products (2022)** (85) (New)
Asia-Pacific	
National Medical Products Administration (NMPA, China)	**Draft guidance: Guidelines for communication and exchange of real-world evidence supporting drug registration applications (2022)** (86) (New)
Taiwan Food and Drug Administration (TFDA)	**Precautions for the use of real-world data/real-world evidence as technical documents for drug review applications (2021)** (87) (New)

Bold font denotes new guidance updates from June 2021 to January 2023.

## Gaps in RWE guidance and future directions to improve RWE use in regulatory decision-making

In our previous publication (1), key gaps and future directions that were identified to further advance in the field of RWE included the need for more detailed guidance on RWD quality and study methods, standardizing the submission process for RWD, international regulatory harmonization, and the impact of the COVID-19 pandemic in accelerating the need for cross-country collaborations. Much of this has been addressed with the recent, more detailed regulatory guidance documents described in the preceding sections. Additionally, funding opportunities, as recently announced by the FDA, also help promote advancements in RWD quality and use, study designs, and developing tools for evaluating different aspects of RWE generation (89). However, regulatory harmonization remains an area for further development with respect to convergence/divergence between regulatory and HTA guidance around RWE and the practical challenges of implementing existing regulatory guidance.

### Implementing RWE guidance into practice

Despite the extensive regulatory guidance released by numerous countries, there is still room for further improvement to fully implement many of the principles into practice. For example, with the release of several guidance documents on data quality principles, more clarity around best practices for sponsors and agencies to assess and document the level of adherence to these principles is needed. This is where further efforts, such as the TransCelerate BioPharma Inc.’s Audit Readiness Tool (75) described earlier, are being directed. Pilot programs, including the FDA’s Advancing RWE Program (26), may also help in further developing the learnings in this area by identifying evidence generation strategies that meet regulatory or post-approval study requirements, developing and promoting agency processes for consistent decision-making, and creating awareness of RWE characteristics to support regulatory decisions. Generating a substantial industry response to such pilot programs is highly recommended to enhance learnings and to inform future guidance. Although new guidance has been issued on data quality standards, there exists a lack of clear and standardized terminology that can be used for all planning and reporting processes to reduce variability, skepticism, and confusion among industry stakeholders (90). Further, industry involvement is also critical in developing a consensus on best practices, especially around data quality, integrity, and analytical methods, which may have a higher likelihood of adoption if consensus reaches a certain threshold.

In addition, although the importance of regulatory authority engagement is clearly described throughout many of the guidance documents, more detailed guidance on best practices for agency engagement on RWE continues to be a point of discussion. TransCelerate BioPharma Inc. has been developing best practices for such engagements on RWE proposals based on member company experience via its Health Authority Engagement Framework (75). Further, there is a lack of comprehensive information on the RWE being submitted to support regulatory decisions and the extent to which this evidence is influencing such decisions. However, regulatory agencies are planning to develop and generate reports for the ecosystem to better understand this.

The US and some other markets require access to patient-level datasets to support marketing applications and efficacy supplements. With access to big data on the horizon, the submission of (properly anonymized) individual patient-level data will aid in credibility and transparency. The data management and sharing policy by the Patient-Centered Outcomes Research Institute (PCORI) is one such example. It requires PCORI research-awardee institutions to generate and preserve research data and document it systematically for ease of data sharing, thereby maximizing the utility and usability of the data (91). Although draft guidance has been released by the FDA in an attempt to harmonize real-world datasets to CDISC format, document areas of infeasibility, and characterize completed transformations, there remains a need for RWD standards for datasets to be established. In addition, the establishment and enforcement of standardized procedures for data derivation and curation would aid in consistency of evidence and should ideally be embedded into the electronically stored health information. In the long term, this could result in different paradigms than the historical clinical trial CDISC format.

Considering all of these, although substantial guidance has been issued, there still remains a key role for experience through individual project proposals and regulatory precedents in informing best practices and the most appropriate regulatory use-cases.

### Regulatory harmonization

With the high number of guidance documents released independently by various countries, harmonization is still just as relevant today as in the last review. Previously, activities by the International Council for Harmonization, Council for International Organizations of Medical Sciences, International Society for Pharmacoeconomics and Outcomes Research, and ICMRA were described (1). Fortunately, these international collaborations continue to be strengthened, in many cases by the experience from the COVID-19 pandemic. In particular, ICMRA facilitated a workshop in June 2022 co-chaired by the EMA, FDA, and Health Canada, in which it issued a formal statement on international collaboration to enable RWE integration into regulatory decision-making. Four key areas for regulatory cooperation were identified, including (a) harmonization of terminologies for RWD/E, (b) regulatory convergence on RWD/E guidance and best practice, (c) readiness to address challenges in public health and prevent emerging health threats, and (d) transparency (92). In addition, the FDA OCE Project Orbis is a potential driver of multinational standards in oncology trial design and best practices, and currently helps provide concurrent cross-agency submission and review of oncology products containing RWE (93). The Society for Translational Oncology’s Global Harmonization of Cancer Trials initiative intends to harmonize universal standards for clinical trial protocols, so as to enhance and support the development of novel strategies for improved treatment and prevention of cancer (94).

### Regulatory vs. HTA convergence/divergence

While RCTs are often the preferred study design by regulatory and HTA bodies, different trial designs are increasingly under consideration in efforts to address the growing unmet medical needs and provide faster access to new and innovative medical therapies for patients. Drug developers are actively seeking to introduce new medicines into the market via a coordinated development program that leverages RWD (as external control groups) to generate evidence that aligns concurrently with the requirements of both regulatory and HTA bodies. In fact, to generate effective medicines that can be approved as well as reimbursed, health care companies feel the need to include HTA requirements early during the development plan (95). Challenges in developing a framework of such synergized evidentiary requirements are not surprising, as regulatory agencies focus on the safety and efficacy of new medicines, while HTA bodies evaluate the relative effectiveness of a new intervention compared to existing ones.

In a survey conducted among stakeholders from the healthcare ecosystem to understand the regulatory and HTA landscape on RWE, pharmaceutical industry stakeholders specified a lack of coordination among regulators and HTA agencies in two major areas: (a) drugs under conditional approval, and (b) medical products associated with clinical uncertainty, such as orphan drugs (95). Additionally, all three stakeholders (industry, regulators, and HTA agencies) frequently perceived divergences in areas related to: (a) acceptable primary endpoints, (b) inclusion of an active comparator arm in trials, and (c) choice and use of surrogate endpoints (95). Other methodological issues to be addressed for improving synchrony were reported to be study design and target patient population (96).

Researchers have proposed that a comprehensive guidance should contain: (a) a well-defined, possibly consensus-based, description of the components involved in each building block pertaining to RWE (namely study design, data sources, analytic methods, transparency and reproducibility, and final report evaluation); (b) a step-by-step procedure that researchers may follow to fulfill regulatory and HTA expectations; (c) a checklist to confirm inclusion of all essential conditions and criteria; and (d) a decision tool for justification, to decision-makers, for adoption of a particular study design (90). Additionally, a few key steps have been recommended by the Duke-Margolis Center for Health Policy to build a robust and aligned ecosystem of RWD for payers and regulators, namely: (a) RWE-driven studies post-regulatory approval of therapies for evidence generation to determine their comparative and/or combined effectiveness, (b) interoperability of data systems for easy use, sharing, and exchange of RWD among multiple systems, (c) linking databases to enhance data analysis potential, (d) standardization of outcomes and endpoints to gage the real-world effectiveness of therapies, (e) supporting collaborations to facilitate data sharing, (f) moving toward robust data collection systems by building on existing databases, and (g) funding and incentives for supporting RWD collection and analysis in the long term (97). The Duke-Margolis Center for Health Policy also recommends a “totality of evidence” approach to generate evidence that is informative not only for regulators, but also for payers (98). The approach suggests that RWE studies should contribute to an evidence package and be considered a part of the totality of evidence when making regulatory decisions.

The last decade has witnessed a significant degree of alignment between regulatory and HTA stakeholders, with a reduction in disparities between regulatory and HTA requirements (95, 96). In the current period of study, we found that several HTA bodies, especially in Europe and Canada, have also started releasing frameworks and guidance focused on RWE. In France, the Haute Autorité de Santé (HAS) published a guide for developing high quality RWE studies on health products, including post-authorization studies, observational studies, and pragmatic trials (99). The guide aims to deliver practical benchmarks pertaining to the methodological aspects for optimizing the level of evidence of these studies and enhancing confidence in the results (99). IMPACT-HTA, the EU’s Horizon-2020 initiative, has also developed recommendations to improve the quality and value of nonrandomized evidence by researchers and HTA bodies (100). The recommendations for researchers include: (a) justifying the need for a nonrandomized study and ensuring that the data are identified via a systematic and transparent process; (b) planning prospective studies to minimize risk of selection bias; and (c) maintaining transparency in reporting data, methods, and results (100). Additional recommendations for HTA bodies include strengthening early scientific advice and investing in resources to efficiently design, analyze, and interpret RWE (100). Furthermore, NICE, the United Kingdom HTA body, released an RWE framework that (a) evaluates and identifies situations where RWD can be applied to reduce uncertainty and improve decision-making, and (b) details best practices to plan, conduct, and report RWE studies to enhance evidence quality and transparency (101). This framework is a part of NICE’s 5-year strategic plan (2021–2026) focused on resolving evidence gaps through the use of RWD and promoting innovative health technology solutions for patients (101). NICE has developed a Data Suitability Assessment Tool (DataSAT), which focuses on data provenance, quality, and reliance, all of which are important considerations for assessing data fitness (101). As per the framework, researchers are expected to justify the choice of RWD source transparently via the use of previously published frameworks, such as Structured Process to Identify Fit-for-Purpose Data (SPIFD) or NICE’s DataSAT (101). NICE hosted a workshop in October 2022 as part of the IMI-EHDEN project highlighting the recent developments in the use of RWE for regulatory decision-making and the implications and opportunities for European HTA agencies to advance adoption of RWE for HTA (102).

Similar to the recently released regulatory guidelines by the FDA, EMA, and MHRA, the guidance issued by HTA bodies aligns with respect to providing recommendations for data quality, reliability, fitness for use, and transparency, so as to promote confidence in RWD studies. This signals an increasing synergy in evidence requirements among regulators and HTA bodies.

Pilot projects to obtain parallel scientific advice on drug development were launched in Australia and Europe involving pharmaceutical companies, HTA agencies, and regulators to address discrepancies in requirements by decision-making bodies (103, 104). In 2022, the EMA launched an initiative to offer parallel joint scientific consultation with the EUnetHTA 21 to generate evidence that is optimal and robust and caters to the requirements of both regulators and HTA bodies (105). To this end, a guidance was published in September 2022 describing the actions and timelines for each party undertaking the parallel EMA/EUnetHTA 21 Joint Scientific Consultation (106). It also discusses practical issues related to the process, such as fees, points of contact, and processing of documents to ensure that all parties are in possession of appropriate document(s) at the correct time (106). The routine inclusion of patients and clinical experts is expected during the joint consultation (106). In Canada, following on its 2019 framework (107), the CADTH announced the expansion of its Scientific Advice Program, which now includes applications for advice on RWE generation plans upon the finalization of protocols for pivotal trials (108). In particular, the jointly developed 2022 RWE-reporting draft guidance from CADTH and Health Canada is a commendable example of regulatory-HTA harmonization and establishes a set of common standards for submissions to both HTA and regulatory agencies (65). The guidance aligns with existing international guidance while providing consideration from the Canadian healthcare perspective.

Indeed, early scientific advice was reported to be a key strategy for evidentiary alignment by agencies and companies, according to a stakeholder survey (95). In fact, it has been observed that manufacturers are more likely to implement changes to the drug development plan based on advice received from both decision-making bodies during such consultations regarding the choice of the primary endpoint and comparator (109). However, a survey emphasized that joint advice discussions were predominantly focused on the regulatory aspect of drug development (95). The consequences of lack of complete alignment could be approval only from one of the authorities (either the regulator or the HTA body). For example, in 2018, axicabtagene ciloleucel received regulatory approval in Europe to treat patients with diffuse large B-cell lymphoma after 2 prior therapies but NICE was unable to recommend its use in the NHS in England at the proposed cost per patient. At that time, this treatment was neither cost-effective nor contained direct comparative evidence to the best supportive treatment care option in England (which was determined to be salvage chemotherapy) (110).

## Conclusion

There have been rapid evolutions in the RWE environment, from the release of general high-level frameworks, to the development and publication of comprehensive, practical guidance documents for RWE practitioners, including industry. This is illustrated by the recent wave of regulatory guidance documents released by several major health authorities focusing on data quality and study methods for RWE. The US and EU continue to lead from the forefront in releasing guidelines to promote the adoption of RWE for regulatory decision-making processes, and China is now also taking a leading role in releasing a number of RWE guidelines in Asia. New frameworks and guidance on RWE are also being issued in many other countries and regions, including the United Kingdom, Taiwan, South Korea, Switzerland, and Australia. However, there is still a global need for the development of guidance using a practical approach to support implementation and adoption. Practical application will rely upon the ability to communicate all aspects of the RWE study design, study conduct, and results clearly and succinctly in situations where large volumes of information often exist. Gaining additional experience and sharing best practices and successful use cases will be important in progressing toward practical data quality reporting standards. Data validation efforts may be expensive and inconsistent in methodologic approach. Multi-stakeholder action will be critical for efficient and robust validation efforts. Additionally, there is also a need for harmonization of guidance across countries and with payers, to expedite the submission and review process with the final goal of access to treatment. Future research evaluating shared aspects and discrepancies among regulatory agencies could advanceharmonization efforts.

## Author contributions

LB: conception, data collection, analysis, interpretation, and critical revision of the article. NR, RK-O, and JC: conception, data collection, and interpretation. JD, SK, and JO’D: conception and data interpretation. FR: data interpretation. All authors contributed to the article and approved the submitted version.

## Footnotes

^1^https://health.ec.europa.eu/system/files/2021-02/pharma-strategy_report_en_0.pdf
