# Supplementary material for: Real-world evidence for regulatory decision-making: updated guidance from around the world
Source: Front Med (Lausanne). 2023 Oct 30;10:1236462. doi: 10.3389/fmed.2023.1236462 (PMC10643567; doi:10.3389/fmed.2023.1236462)
Supplement: Supplementary file 1 [file Table_1.docx]

Supplementary Material

Real-World Evidence for Regulatory Decision-Making: Updated Guidance from Around the World

# Supplementary Table

**Table 1:** Third-party initiatives for advancement in real-world evidence (RWE) development and use.

| **Key Regulatory Elements Pertaining to RWE** | **Third-Party Initiatives** |
| --- | --- |
| **Regulatory RWE frameworks** | Duke-Margolis Center for Health Policy RWE Collaborative (2022) (1)  European Health Data & Evidence Network (EHDEN) Academy (2) |
| **RWD quality guidance** | University of California, San Francisco-Stanford University Center of Excellence in Regulatory Science, and Innovation (CERSI) and the FDA: OneSource Project (2022) (3)  FDA Oncology Centre of Excellence (OCE) and Regan Udall Foundation: Quality Characteristics and Assessment of Real-world Data (QCARD) initiative (2022) (4)  ICARE data^®^ (Integrating Clinical Trials and Real-world Endpoints) project (5)  TransCelerate BioPharma Inc.’s Audit Readiness Tool (6)  HARMONY Alliance Big Data Platform (7)  Clinical Data Interchange Standards Consortium (CDISC) (8)  EUnetHTA Joint Action 3 Registry Evaluation and Quality Standards (REQueST) Tool (9) |
| **Study methods guidance** | RCT DUPLICATE (Randomized, Controlled Trials Duplicated Using Prospective Longitudinal Insurance Claims: Applying Techniques of Epidemiology) (10)  Duke-Margolis Point-of-Care Clinical Trials: Integrating Research and Care Delivery (11)  Friends of Cancer Research Characterizing endpoints for real-world data capture (12)  GetReal Academy (13) |

**References**

1. Duke-Margolis Center for Health Policy. Real-World Evidence Collaborative (2022) [October, 2022]. Available from: <https://healthpolicy.duke.edu/projects/real-world-evidence-collaborative>.

2. EHDEN. EHDEN Academy (2022) [February, 2023]. Available from: <https://academy.ehden.eu/>.

3. U.S. Food & Drug Administration. Source Data Capture from Electronic Health Records (EHRs): Using Standardized Clinical Research Data (Onesource Phase I) (2021) [July, 2022]. Available from: <https://www.fda.gov/science-research/advancing-regulatory-science/source-data-capture-electronic-health-records-ehrs-using-standardized-clinical-research-data>.

4. U.S. Food & Drug Administration. Driving Medical Product Development (2022) [September, 2022]. Available from: <https://www.fda.gov/about-fda/2021-oce-annual-report/driving-medical-product-development>.

5. Collaborative TSHR. Icaredata® Project [September, 2022]. Available from: <http://icaredata.org/>.

6. Transcelerate Biopharma Inc. Real World Data (2022) [July, 2022]. Available from: <https://www.transceleratebiopharmainc.com/initiatives/real-world-data/>.

7. Harmony Alliance. Big Data Platform (2021) [May, 2022]. Available from: <https://www.harmony-alliance.eu/bigdata-platform/big-data-platform>.

8. Facile R, Muhlbradt EE, Gong M, Li Q, Popat V, Petavy F, et al. Use of Clinical Data Interchange Standards Consortium (CDISC) Standards for Real-World Data: Expert Perspectives from a Qualitative Delphi Survey. *JMIR Med Inform* (2022) 10(1):e30363. Epub 2022/01/28. doi: 10.2196/30363.

9. Allen A, Patrick H, Ruof J, Buchberger B, Varela-Lema L, Kirschner J, et al. Development and Pilot Test of the Registry Evaluation and Quality Standards Tool: An Information Technology-Based Tool to Support and Review Registries. *Value Health* (2022). Epub 2022/03/13. doi: 10.1016/j.jval.2021.12.018.

10. Franklin JM, Patorno E, Desai RJ, Glynn RJ, Martin D, Quinto K, et al. Emulating Randomized Clinical Trials with Nonrandomized Real-World Evidence Studies: First Results from the Rct Duplicate Initiative. *Circulation* (2021) 143(10):1002-13. Epub 2020/12/18. doi: 10.1161/CIRCULATIONAHA.120.051718.

11. Duke-Margolis Center for Health Policy. Point-of-Care Clinical Trials: Integrating Research and Care Delivery (2022) [May, 2022]. Available from: <https://healthpolicy.duke.edu/publications/point-care-clinical-trials-integrating-research-and-care-delivery>.

12. Friends of Cancer Research. 2021 Scientific Report: Regulatory Advancements for Patients (2021) [May, 2022]. Available from: <https://friendsofcancerresearch.org/wp-content/uploads/2021-Friends-of-Cancer-Research-Scientific-Report.pdf>.

13. GetReal Institute. GetReal Institute: Homepage [February, 2023]. Available from: <https://www.getreal-institute.org/>.
